# Corrigendum

**DOI:** 10.1002/rmb2.12081

**Published:** 2018-01-16

In ref. 1, an error was published in the title of the article on page 268.

Weight reduction by using a formula diet recovers menstruation in obese patients with an ovulatory disorder.

The title was incorrect and should be:

Weight reduction using a formula diet recovers menstruation in obese patients with an ovulatory disorder.

The title has also been corrected online on 2nd November 2017.

The author apologizes for this error.
